# Using cellzilla for plant growth simulations at the cellular level

**DOI:** 10.3389/fpls.2013.00408

**Published:** 2013-10-16

**Authors:** Bruce E. Shapiro, Elliot M. Meyerowitz, Eric Mjolsness

**Affiliations:** ^1^Department of Mathematics, California State UniversityNorthridge, CA, USA; ^2^Biological Network Modeling Center, CaltechPasadena, CA, USA; ^3^Division of Biology, Howard Hughes Medical Institute, CaltechPasadena, CA, USA; ^4^Department of Computer Science, University of CaliforniaIrvine, CA, USA

**Keywords:** mathematical model, computational model, software, meristem, cellerator, cellzilla, wuschel, clavata

## Abstract

Cellzilla is a two-dimensional tissue simulation platform for plant modeling utilizing Cellerator arrows. Cellerator describes biochemical interactions with a simplified arrow-based notation; all interactions are input as reactions and are automatically translated to the appropriate differential equations using a computer algebra system. Cells are represented by a polygonal mesh of well-mixed compartments. Cell constituents can interact intercellularly via Cellerator reactions utilizing diffusion, transport, and action at a distance, as well as amongst themselves within a cell. The mesh data structure consists of vertices, edges (vertex pairs), and cells (and optional intercellular wall compartments) as ordered collections of edges. Simulations may be either static, in which cell constituents change with time but cell size and shape remain fixed; or dynamic, where cells can also grow. Growth is controlled by Hookean springs associated with each mesh edge and an outward pointing pressure force. Spring rest length grows at a rate proportional to the extension beyond equilibrium. Cell division occurs when a specified constituent (or cell mass) passes a (random, normally distributed) threshold. The orientation of new cell walls is determined either by Errera's rule, or by a potential model that weighs contributions due to equalizing daughter areas, minimizing wall length, alignment perpendicular to cell extension, and alignment perpendicular to actual growth direction.

## Introduction

In recent years, there has been much interest in accurate multi-scale models of morphogenesis. Due to the various levels of complexity and the wide variety of tissue, there has necessarily been a trade-off between generality and specificity; an excellent review is given by Koumoutsakos et al. (2011). Of the most interest to plant biologists, perhaps, are platforms that describe the high-level structure of complete organisms, systems, or organs such as a meristem or sepal. Very few general purpose tools exist at all, and even fewer are specific to plants. L-System based tools, the best known being L-Studio (Karwowski and Prusinkiewicz, 2004), were among the first; they are ideally suited to branching structures because they are based on formal language theory (a grammar based on axioms, a short alphabet, strings derived from that alphabet based on specific production rules that are tuned to branching). L-system rules have been embedded in higher level languages such as C [specifically, *cpfg*, (Prusinkiewicz and Lindenmayer, 1990)] to allow the encoding of models and the description of geometrical and topological relationships. The Virtual Plant's OpenAlea Platform (Pradal et al., 2008) provides a general-purpose collection of simulation modules that use Python as the primary scripting language and allows L-systems to be incorporated (Boudin et al., 2012).

Outside of plant biology, much of the interest has focused on grid-based models such as the Cellular Potts Model (CPM) (Graner and Glazier, 1992), which discretize to the desired sub-cellular level of detail as collections of entities driven by particle-particle interactions; and finite element models (FEM) which instead discretize to a sufficiently fine web-work of straight lines under the influence of mechanical forces. Because of the molecular level of detail of these methods they are often primarily stochastic in nature (Gilllespie, 1976). There has been some effort to provide general purpose platforms; examples include MCell (Stiles and Bartol, 2001), designed originally to simulate the synaptic junction; Smoldyn (Andrews, 2012), primarily emphasizing nano-scale specificity; ChemCell (Plimpton and Slepoy, 2005), which models protein networks within cells; and CompuCell3D (Cickovski et al., 2007) which focuses on cellular function.

Of particular interest is the use of rule-based models, e.g., BioNetGen (Faeder et al., 2005, 2009) and NFSIM (Sneddon et al., 2011). In a rule-based model, molecules are considered structured objects and their interactions are described by rules for transforming these objects. Rule based models are interesting because every process that occurs within a biosystem, such as chemical and physical interactions and growth, development, and cell division and death, can be described by rules. Similarly, Dynamical Grammars (Mjolsness and Yosiphon, 2006; Yosiphon, 2009; Mjolsness, 2013) define models in terms of operator algebras of stochastic processes, with simulation algorithms derived from the composition and expansion of time-evolution operators.

At an intermediate level of complexity there are tools that describe tissue at the multicellular level, treating each cell as a well-mixed compartment. Cells can be described either as simple point (or spherical) objects connected by breakable springs (Jönsson et al., 2004, 2005a,b; Mjolsness, 2006), or at some level of geometric complexity with springs between polygonal vertices (Rudge and Haseloff, 2005; Sahlin and Johnsson, 2010), as is currently done by Cellzilla. The VirtualLeaf (Merks et al., 2011) provides an interesting hybrid that uses the CPM in combination with mechanical springs with a Markovian relaxation algorithm to describe tissue growth. Cellzilla has the distinction among these software implementations of extending the collection of Cellerator Arrows to include multicellular interactions (Shapiro et al., 2003).

## Materials and methods

### Cellerator reactions

Interactions are described in terms of Cellerator arrows (Shapiro et al., 2003); the basic canonical form is {*X* → *Y*, *k*}. When several species are interacting the arrow expression is written as

(1) $$e_{1}X_{1}+e_{2}X_{2}+\cdots \to f_{1}Y_{1}+f_{2}Y_{2}+\cdots$$

where the *e*_*i*_ and *f*_*j*_ are stoichiometries. Each reactant is converted to a single term in a differential equation according to mass action kinetics:

(2) $$\frac{dU_{j}}{dt}=(f_{j}-e_{j})kX_{1}^{n_{1}}X_{1}^{e_{1}}X_{2}^{e_{2}}\cdots$$

where *e*_*j*_ and *f*_*j*_ are the stoichiometries of species *U*_*j*_ in the reaction on the left and right hand side of the reaction, respectively, and *k* is the rate constant of the reaction. More complex expressions can be built from this canonical form to represent exact mass action descriptions of multi-species complex enzymatic reactions, as summarized in Table 1, and numerous regulatory approximations such as Michaelis-Menten, Hill functions, and MWC equations, as summarized in Table 2. Furthermore, a large number of exact enzymatic expansions have been implemented using the basic canonical form via the KMech toolbox, including BiBi, BiTer, BiUni, MulS, OrderedBiBi, OrderedBiUni, PingPong, PingPongTerTerF, PingPongTerTerR, RandomBiBi, TerBi, TerTer, UniBi, and UniUni reactions (Yang et al., 2005).

**Table 1:** **Examples of Cellerator mass action arrow form expansion, from the base from *A* → *B***.

Arrow form^*^	**Expansion**
e_1_X_1_ + e_2_X_2_ + … ⇄	e_1_X_1_ + e_2_X_2_ + … → f_1_Y_1_ + f_2_Y_2_ + …
f_1_Y_1_ + f_2_Y_2_ + …	f_1_Y_1_ + f_2_Y_2_ + … → e_1_X_1_ + e_2_X_2_ + …
$X\overset{ℰ}{⇄}Y$	X + ℰ ⇄ X_ℰ
	X_ℰ ⇄ Y + ℰ
$X\underset{ℱ}{\overset{ℰ}{⇄}}Y$	$X\overset{ℰ}{⇄}\text{Y and Y}\overset{ℱ}{⇄}X$
$X\overset{ℰ}{⇌}Y$	X + ℰ ⇄ X_ℰ
	X_ℰ ⇄ Y_ℰ
	Y_ℰ ⇄ Y + ℰ
$\overset{ℰ}{X⇄Y⇄Z⇄\cdots }$	$X\overset{ℰ}{⇄}Y,\text{ Y}\overset{ℰ}{⇄}Z,\ldots$
$\underset{ℱ}{\overset{ℰ}{X⇄Y⇄Z\cdots }}$	$X\underset{ℱ}{\overset{ℰ}{⇄}}Y,\text{ Y}\underset{ℱ}{\overset{ℰ}{⇄}}Z,\ldots$

* *The complete syntax of an arrow form is {arrowform, k_1_,…} where k_1_,… is a sequence of numeric or symbolic rate constants. The subscripts e_i_ and f_j_ are stoichiometries*.

**Table 2:** **Cellerator user defined, regulatory, and enzymatic arrow forms**.

**Arrow form^a,b^**	**Typical ODE Term^c^**
{**e** · **X** ⇒ **f** · **Y**, g(**X**)}	[X_i_]′ = −e_i_g(**X**), [Y_i_]′ = f_i_g(**X**)
$\left\{X\overset{ℰ}{\mapsto }Y,\text{ Hill}[v,\;n,\;K,\;a,\;T]\right\}$	${\left[Y\right]}^{\prime }\text{=}\frac{v[ℰ]\;{\left(a\text{ + }T\;\cdot \;[X]\right)}^{n}}{K^{n}\text{ + }{\left(a\text{ + }T\;\cdot \;[X]\right)}^{n}}$
$\left\{X\overset{ℰ}{\mapsto }Y,\text{ GRN}[v,\;T,\;n,\;h]\right\}$	${\left[Y\right]}^{\prime }\text{=}\frac{v[ℰ]}{\text{1+}\exp\left(-h-T\;\cdot \;{\left[X\right]}^{n}\right)}$
$\left\{X\overset{ℰ}{\mapsto }Y,\text{ SSystem}[\tau ,\;C_{\text{+}},\;C_{-},\;n^{+},\;n^{-}]\right\}$	${\left[Y\right]}^{\prime }=\frac{\left[ℰ\right]}{\tau }\left\{C_{+}\prod_{i}X_{i}^{n_{i}^{+}}-C_{-}\prod_{i}X_{i}^{n_{i}^{-}}\right\}$
$\left\{X\overset{ℰ}{\mapsto }Y,\text{ NHCA}[v,\;\{T^{+},\;T^{-}\},\;n,\;m,\;k]\right\}$	${\left[Y\right]}^{\prime }\text{=}\frac{v[ℰ]\prod_{i}{\left(\text{1+}T_{i}^{+}{\left[X\right]}_{i}^{n_{i}}\right)}^{m}}{k\prod_{i}{\left(\text{1+}T_{i}^{-}{\left[X\right]}_{i}^{n_{i}}\right)}^{m}\text{+}\prod_{i}{\left(\text{1+}T_{i}^{+}{\left[X\right]}_{i}^{n_{i}}\right)}^{m}}$
{{**X, Y**} ⇒ rational[**a, d, m, n**]}	${\left[Y\right]}^{\prime }d\text{=}\frac{a_{\text{0}}\text{ + rest}\left(a\right)\;\cdot \;{\left[X\right]}^{n}}{d_{\text{0}}\text{ + rest}\left(d\right)\;\cdot \;{\left[X\right]}^{n}}$
{**X** ↦ Y, USER[*v*, **T**, **n**, *h*, *f*]}	[Y]′ = *vf*(*h* − **T** · [**X**]^n^)
$\left\{X\overset{ℰ}{\Rightarrow }Y,\text{ MM}[\text{K},\text{ v}]\right\}$	${\left[Y\right]}^{\prime }\text{ = }-{\left[X\right]}^{\prime }\text{ = }\frac{v[ℰ][X]}{K\text{ + }[X]}$
$\left\{X\overset{ℰ}{\Rightarrow }Y,\text{ MWC}[k,\;n,\;c,\;L,\;K]\right\}$	${\left[Y\right]}^{\prime }e\text{=}-{\left[X\right]}^{\prime }\text{= }k[ℰ]\frac{\alpha {\left(\text{1+}\alpha \right)}^{n-\text{1}}\text{+}L\alpha \text{c}{\left(\text{1+}\alpha c\right)}^{n\;-\text{1}}}{{\left(\text{1+}\alpha \right)}^{n}\text{+}L{\left(\text{1+}\alpha c\right)}^{n-\text{1}}}$

*^a^The catalyst species ℰ is optional and may be omitted*.

*^b^Boldface quantities may be either scalars or vectors. Vectors enclosed by curly brackets. The notation **v** = **p^q^** means a vector **v** with components v_i_ = p^qi^_i_*.

*^c^If the catalyst is omitted replace*[ℰ] *with 1*.

*^d^In* rational, *rest(**x**) is the vector **x** with its first element removed*.

*^e^In MWC, α = [X]/K*.

Cellzilla is implemented in Mathematica and is invoked using the standard notebook interface. The details of numerical integration are normally hidden from the user, e.g., models (such as (30)–(32)) are submitted to the kernel by the Cellzilla Grow command and Cellerator run along with a list of simulation control parameters and initial conditions. These front-end commands directly invoke Mathematica's NDSolve. Normally, the default solver in NDSolve is chosen, unless the user selects otherwise; any control option for NDSolve may be passed along by Grow command. For ordinary differential equations, NDSolve switches between a non-stiff Adams method and a stiff Gear Backward Differentiation formula. However, users may optionally change the parameters or choose another solver.

### Tissue description

Tissues are described by a polygonal lattice, with each lattice cell representing one biological cell. The Tissue data structure consists of: (1) a vertex list **V**, where each vertex **V**_*i*_ is an (*x*, *y*) pair; (2) an edge list **E**, where each edge **E**_*k*_ = (*i*_*k*_, *j*_*k*_) is a pair of integers giving indices of vertices at the endpoints of the edge; and (3) a list of cells **C**, where each cell **C**_*k*_ = {*k*_1_, *k*_2_, *k*_3_, …} is an ordered list of edge indices. Externally the tissues may be saved (either read or written) as CSV files, either as lists of vertices, edges, and cells, or in a flattened versions with the edges omitted and the cells represented as ordered sequences of vertices. Internally the edges are always reconstructed since it is more efficient computationally to always have the edge information available, but it is user taste which I/O format is used. It was decided to use the CSV format for these files because of the wide availability of parsing tools and the ease of human readability should that be necessary.

Species in different cells are referenced by an index; e.g., the reaction X[17] → Y[17] takes place in cell 17. When a reaction network is expanded in every cell in the system it is not necessary to repeat this manually, as this is done automatically. Constituents in different cells can interact in the following ways: (a) diffusion; (b) action at a distance; (c) transport across the cell wall. Each of these may be specified in the model by one of the additional Cellzilla Arrow forms listed in Table 3. Such interactions are allowed to depend on a function *f*(*i*, *j*, *k*) that depends on the properties of the constituents of the cells (*i* = present cell number, *j* = connecting cell numbers) and cell wall (*k*) between cells *i* and *j*. The basic single cell models may be input and output as SBML files using the Cellerator/MathSBML extensions for SBML (Hucka et al., 2003; Shapiro et al., 2004).

**Table 3:** **Additional Cellzilla arrow forms not recognized by Cellerator**.

Arrow form^*^	**Description**
{X → X, Diffusion[P_I_, P_O_]}	Diffusion of X through the tissue. P_I_ is the permeability of internal cell walls; the optional P_O_ is the permeability of tissue boundary cell walls. Each may be specified as **f[i, j, k]** where *i*, *j*, *k* are cell and wall indices.
{X → X, Transport[fout, fin]}	Controlled transport of **X** across the cell wall.
{X ↦ Y, IGRN[*v*, **T**, **n**, *h*]}	${\left[Y[j]\right]}^{\prime }\text{=}\frac{v}{\text{1+}\exp(-h-T\;\cdot \;{\left[X[i]\right]}^{n})}$
{cell → cell, Grow[…]}	Specification of cell growth parameters: Pressure, growth rate, spring constant.
{cell → cell + cell, *model*[…]}	Specification of cell division model and parameters.

* *Except for the* IGRN *the arrows here are longer versions of the right-pointing arrows used by the canonical Cellerator mass-action expansion*.

Diffusion across cell boundaries is implemented according to Fick's law, so that the flux *J* through any membrane (e.g., in molecules/(cm^2^-s)) with diffusion constant *D* (e.g., in cm^2^/s) is

(3) $$J=-D\frac{\partial X}{\partial x}$$

Defining the membrane permeability as β = *D*/δ (e.g., in cm/s), where δ the membrane (or wall) thickness gives

(4) $$J=-\beta \delta \times \frac{\Delta X}{\delta }=-\beta \Delta X$$

where Δ*X* is the concentration difference across the membrane. Let cell *i* have area *A*_*i*_ and depth *d* (orthogonal to the simulation); the the volume of cell *j* is *V*_*j*_ = *A*_*j*_*d*, and the area of the cell wall between cell *i* and cell *j* is ℓ_*k*_*d* where ℓ_*k*_ is the length of the wall between the cells. The flux across wall *k* into cell *i* is (for constant area):

(5) $$J=\left(\frac{d[X_{i}]}{dt}\times A_{i}d\right)\times \left(\frac{1}{\ell_{k}d}\right)=\frac{A_{i}}{\ell_{k}}\frac{d[X_{i}]}{dt}$$

Therefore

(6) $$\frac{d[X_{i}]}{dt}=\frac{\beta \ell_{k}}{A_{i}}\left(\left[X_{j}]-[X_{i}\right]\right)$$

Rather than implementing this equation directly, it is implemented as equivalent Cellerator reactions

(7) $$\left\{X[i]⇄\emptyset ,\frac{\ell_{k}}{A_{i}}\text{​}f(i,j,k),\;\frac{\ell_{k}}{A_{i}}\text{​}f(i,j,k)X[j]\right\}$$

where *f* is the input concentration-dependent permeability. Diffusion is specified to Cellzilla by incorporating arrows of the following form into the model:

(8) {X→X,Diffusion[f[i,j,k]]}

where f is either a Mathematica pure function or a function that has been defined previously in the simulation.

Action at a distance is technique for describing the way constituents in cell *i* can affect constituents in another cell *j* without specifying any of the intermediate reactions. We restrict action at a distance to adjacent cells with Cellerator GRN-like reactions written as {X ↦ Y, IGRN[*v*, β, *n*, *h*]} which means that constituent *X*_*i*_ affects constituent *Y*_*j*_ in neighboring cell *j* according to (Mjolsness et al., 1991)

(9) $$\frac{d[Y_{j}]}{dt}=\frac{v}{1+e^{-h-\beta [X_{i}]}}$$

Facilitated membrane transport from cell *j* to cell *i* is described by the equations

(10) $$\frac{d[X_{i}]}{dt}=\frac{\ell_{k}}{A_{i}}\text{​}\left(f_{in}\text{​}(i,j,k)-f_{out}\text{​}(i,j,k)-f_{in}\text{​}(j,i,k)+f_{out}\text{​}(j,i,k)\right)$$

where *f*_*out*_(*i*, *j*, *k*) is the positive outward molecular flux from *i* through edge *k*, to *j*, and *f*_*in*_(*i*, *j*, *k*) is the positive inward molecular flux to cell *i* through edge *k*, originating from cell *j*. In most cases one will only want to specify one of *f*_*out*_ or *f*_*in*_. These are implemented internally via the Cellerator reactions

(11) $$\left\{X[i]\Rightarrow \emptyset ,(\ell_{k}/A_{i})(f_{out}\text{​}[i,j,k]+f_{in}\text{​}[j,i,k])\right\}$$

(12) $$\left\{\emptyset \Rightarrow X[i],(\ell_{k}/A_{i})(f_{in}\text{​}[i,j,k]+f_{out}\text{​}[j,i,k])\right\}$$

Transport reactions of this sort are specified to Cellzilla by including arrows of the form

(13) {X→X,Transport[fout,fin]}

where the function fout should be set to zero if only f_in_ is utilized.

### Growth model

Cellzilla implements two types of time-dependent simulations: static, and growing. In static simulations the shape of the tissue and its component parts do not change but its constituents are allowed to vary as described previously. In a growing tissue, the shape of the cells are also allowed to evolve with time. Cell growth is described by associating a Hooke's law spring potential of the form

(14) $$V_{ij}=\frac{1}{2}\sum k_{ij}{\left(\delta_{ij}-\ell_{ij}\right)}^{2}$$

with the edge connecting vertices **x**_*i*_ and **x**_*j*_. Here δ_*ij*_ is an equilibrium length assigned to the edge, ℓ_*ij*_ is the actual length, and *k*_*ij*_ is a constant. When the wall is under compression (so that δ_*ij*_ > ℓ_*ij*_) there will be a force, acting along the length of the edge, pushing the two vertices apart; when the wall is extended (δ_*ij*_ < ℓ_*ij*_), the force will tend to pull the vertices toward one-another. The magnitude of this force is equal to the negative gradient of *V*_*ij*_. In addition, a pressure *P*_*a*_ associated with each cell *a* is, is applied outward at each vertex. The net force on each vertex is proportional to each wall incident on that vertex and, and is split evenly between the vertices. Then equation of motion for vertex **x**_*i*_ is then

(15) $$\frac{dx_{i}}{dt}=-\sum_{j}k_{ij}{\hat{x}}_{ij}(\ell_{ij}-\delta_{ij})+\frac{1}{2}\sum_{j,\;a}P_{a}n_{ij,\;a}\ell_{ij}$$

where ${\hat{x}}_{ij}$ is a unit vector pointing from **x**_*i*_ to **x**_*j*_. The sum in the first term is over all the neighbors *j* of vertex *i*. In the second term, **n**_*ij, a*_ is an outward pointing unit-normal vector from cell *a*, normal to edge ℓ_*ij*_, and the sum is over all neighbors *j* of *i* and and over all cells *a* in incident at vertex *i*. The pressure force will cause the springs to extend, simulating cell growth. The resting length is allowed to increase linearly at a rate proportional to the extension beyond resting length,

(16) $$\frac{d\delta_{ij}}{dt}=\mu_{ij}\Theta (\ell_{ij}-\delta_{ij})$$

where

(17) $$\Theta (x)=\frac{1}{2}(x+|x|)=\{\begin{array}{ll} x & \text{if }x\geq 0\\ 0 & \text{otherwise} \end{array}$$

If the spring is not extended, then growth does not occur. Equations (15) and (16) capture the phenomenological behavior observed in nature by both plant and animal cells. Pressure drives cell expansion; as pressure increases, cells expand more quickly. The growth rate is limited by the spring force. The dynamics of (15) can be solved exactly for a square cell to give

(18) $$\frac{1}{L}\frac{dL}{dt}=k\left(\frac{P}{k}-\frac{2\Delta }{L}\right)$$

where *L* is the cell perimeter and Δ=∑_*i*_δ_*i*_ is the sum of the resting lengths. Consequently, when μ_*ij*_ = 0 in (16), the sigmoidal growth pattern of plant cells is observed [e.g., when extensibility decreases linearly over time and osmotic pressure is held constant, as in Figure 1 of Lockhart (1965)]. Comparing (18) to the Lockhart equation ℓ′/ℓ = Φ(*P* − *P_E_*) we see that the *k* and δ are parameters that can tuned to fit effective extensibility Φ and yield pressure *P*_*E*_; for constant *P* and *k* sigmoidal behavior can be tuned for *P* < 2*k* in square cells. Additionally, allowing μ > 0, along with the spring force produces a more general growth model that is more generally applicable, not just in plant tissue, as the springs can be cut beyond a specified threshold, thereby removing cell-cell interactions (Shapiro and Mjolsness, 2001).

Let *r* be the index of the edge connecting vertices *i* and *j*, so that δ_*r*_ and ℓ_*r*_ are short notations δ_*ij*_ and ℓ_*ij*_. The spring dynamics of (16) are implemented internally as

(19) $$\left\{\emptyset \to x[i,p],\;\sum_{\text{Neighbors(i)}}k[r](x[j,p]-x[i,p])\left(1-\delta_{r}/\ell_{r}\right)\right\}$$

where **x**[i, p] is the *p*th Cartesian component (*p* = 1, 2 for *x* or *y*) of vertex **x**_*i*_, and k[r] is a spring constant whose value may depend on the properties (e.g., constituents of) the cells abutting edge *r*. Similarly, the growth of edge δ_*r*_ described by (16) is implemented internally as the Cellerator reactions

(20) $$\left\{\emptyset \to \delta_{r},\mu [p,q,r](\ell_{r}-\delta_{r})\right\}$$

where p and q are the indices of the cells that about edge *r*. The pressure force in (15) on vertex *x*_*i*_ due to cell *a* on the edge connecting vertices **x**_*i*_ and **x**_*j*_ is implemented internally as

(21) $$\left\{\emptyset \to x[i,k],\frac{1}{2}P[a]n[a,i,j,k]\right\}$$

where k = 1, 2 (to indicate *x* or *y* Cartesian component); P[a] is the pressure in cell a; and n[a, i, j, k] is the k^*th*^ component of a unit normal vector to edge ℓ_ij_ pointing outwards from cell a.

Cell growth is specified in a Cellzilla model by a reaction of the form

(22) {cell→cell,Grow[⋯]}

where the arguments to Grow specify growth parameters such as the dependence of k, P, and μ on cell constituents (see Table 3). Chemical concentrations change during growth in each cell occur even in the absence of reactions. If there are *n* molecules of *X* in volume *V* then [*X*]′ = (*n/V*)′ = (*Vn*′ − *nV*′)/*V*^2^ = *n*′/*V* − [*X*]*V*′/*V*. The second term gives the correction in [X]' due to volume changes.

### Cell division

Division occurs when a cell's area passes a threshold. Upon birth, each cell is assigned a threshold that is distributed normally (with the mean μ and standard deviation σ as optional control parameters). Chemical concentrations are distributed equally between the child cells (so that the chemical amounts are proportional to cell area). A single (linear) near cell wall is placed according to one of two user-selectable model: the standard modern interpretation of (Errera, 1888) and a potential model. In the modern interpretation of Errera's rule, the shortest wall that divides the two cells in half (by area) is chosen. [Technically, this is not Errera's rule, which only defines the shape of the cell wall, once the endpoints are already know; however, the area-equalization constraint is typically added to provide this boundary condition. (Smith, 2001; Besson and Dumais, 2011; Prusinkiewicz and Runions, 2012)] In the potential model (2010, 2010) a function

(23) $$V(\theta_{1},\theta_{2})=\sum_{i}w_{i}V_{i}(\theta_{1},\theta_{2})$$

is minimized over the central angles θ_1_ and θ_2_. These give the central angles of the end points of the new wall measured from the cell centroid. Here **w** is a weight vector, and *V*_*i*_ represents each contributor to cell division, where *i* ∈ {*A, L, e, g*}, as described in the below.

The area potential *V*_*A*_ is minimized when the cell divides in half; if the daughter cells have areas *A*_1_ and *A*_2_, respectively, then we define

(24) $$V_{A}={\left(\frac{A_{1}-A_{2}}{A}\right)}^{2}$$

The function is squared to improve computational stability near the minimum (which would otherwise have a non-differentiable corner there). The disadvantage of this potential is that it does not have a unique global minimum, i.e., any line of cell division will that divides the area in half will give a value of zero. Thus, the area potential must be tempered by either an additional potential function (such as the perpendicularity and/or length potential) or an additional heuristic to select the desired minimum value that does not require a unique minimum (e.g., randomly select division direction from amongst all equivalent minima).

The length potential *V*_*L*_ will be minimized when the new cell wall is most closely aligned with the shortest possible diameter *d*_*min*_ that this, the shortest line segment dividing the cell passing through the cell center. If *d* is the length of the new cell wall, then *V*_*L*_ is given by

(25) $$V_{L}=\frac{{\left(d-d_{min}\right)}^{2}+\epsilon_{L}\Delta^{2}}{{\left(d+d_{min}\right)}^{2}}$$

where Δ is the shortest distance between the new wall and the cell center, and ϵ_*L*_ is a tunable parameter.

For *V*_*e*_ and *V*_*g*_ we define a perpendicularity potential *V*_⊥_(**v**) that is minimized when the new wall is perpendicular to particular unit vector **v**. Let **W** be a unit vector parallel to the new wall. Then

(26) $$V_{⊥}(v)=v\cdot W+\frac{\epsilon_{⊥}\Delta }{d}$$

where ϵ_⊥_ is a parameter. Letting **e** be the direction of maximal cell extension and **g** be the direction of maximal cell growth, we then define

(27) $$V_{e}=V_{⊥}(e)$$

(28) $$V_{g}=V_{⊥}(g)$$

so that *V*_*e*_ and *V*_*g*_ are minimized when **W** is most nearly perpendicular to the directions of maximal extension and maximal cell growth, respectively. The direction of maximal extension is taken as the unit eigenvector corresponding to the larger eigenvalue of the covariance matrix **M** = (**X**^*T*^**X**)/(*n* − 1) where *n* is the number of cell vertices; and **X** = [**x** − **x**_*c*_|**y** − **y**_*c*_], where **x** and **y** are column vectors of cell vertex coordinates {(*x*_1_, *y*_1_), …, (*x*_*n*_, *y*_*n*_)} and **x**_*c*_ and **y**_*c*_ is their mean. The direction of instantaneous maximal growth is found in the same manner, using the velocities of the vertices. The covariance matrix **M** is calculated using the Mathematica function Covariance.

Cell division is specified in a Cellzilla model with the arrow

(29) {cell→cell+cell,model[⋯]}

where model is either ErreraModel or Potential, and the arguments specify the threshold variable, mean, standard deviation, and weight vector (for the potential model). A more generalized version of this notation has been introduced by Yosiphon (2009).

## Results

### Templates and the brusselator

Cellzilla has variety of shapes that can be used for basic simulations such as rectangular and hexagonal arrays, as well as circular, semicircular and parabolic templates that can be populated with randomly placed Voronoi centers. Alternatively the user can supply a template of his or her own consisting either of cell enters (in which case the walls will be interpolated with a Voronoi algorithm) or cell walls as described previously. Here we present the use of several of these templates (hexagonal, Voronoi, and user-supplied) to implement a common reaction-diffusion system.

**Figure 1:**
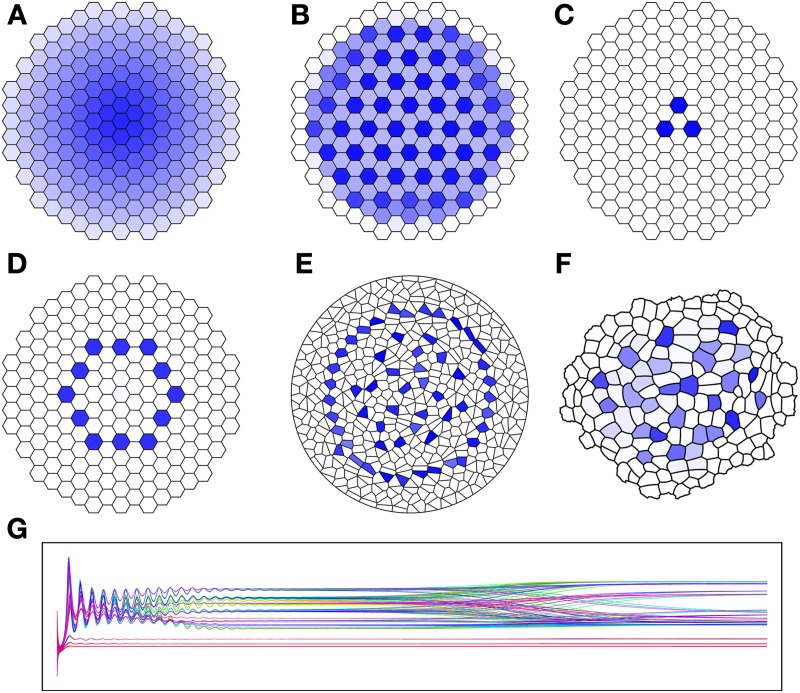
**Results of Brusselator simulation showing affect of ratio of diffusion constant and geometry on simulation. (A–F)** The concentration of species *A* in the brusselator (30) is shown, with higher concentration given in dark blue, and zero concentration in white. **(A)** D_A_ = D_B_ =1; **(B)** D_A_ = 0, D_B_ = 0.1; **(C)** D_A_ = 0, D_B_ = 1; **(D–F)** all use D_A_ = 0, D_B_ = 0.5 on different cellular teimplates. **(G)** Time course of the concentration of species *A* for all cells in simulation B (total of 199 cells). Each curve gives the concentration for different cell. Simulation E uses a Voronoi template of 500 randombly placed centers, and F uses a actual *Arabodopsis* meristem L1 segmentation. Parameters: a = 0.1, β = 0.1, c = 0.1.

The Brusselator (Prigogine and Lefever, 1968) is frequently cited in mathematical modeling because it consists of a system of chemical reactions that in the appropriate parameter regime will maintain sustained oscillations. When combined with diffusion such a system can also be used to establish a wide variety of interesting patterns such as stripes, spirals, and central maxima. The establishment of these different patterns depends on the choice of system geometry, boundary conditions, and parameter values. A diffusible Brusselator is easily implemented in Cellzilla with

(30) $$\begin{array}{l} \{\{\emptyset ⇄A,a,\beta \},\{2A+B\to 3A,c\},\{A\to B,b\},\\ \;\{A\to A,Diffusion[D_{A},D_{A}]\},\\ \;\{B\to B,Diffusion[D_{B},D_{B}]\}\} \end{array}$$

where a, β, b, c, D_A_ and D_B_ are tunable parameters. As illustrated in Figure 1, the ratio of the diffusion constants will change the number of maxima achieved. In the second row of the figure we see that the geometry is also significant. While the qualitative features of each of the results D-F are identical, their symmetry becomes more and more broken as the symmetry of the template becomes lost. With the exception of the diffusion constants, all other parameters were identical through each of these simulations. We are particularly interested in the parameter set shown in Figure 1A, because it can be used as described in the following section to establish an organizing center for simulation of the WUS/CLV network.

### Establishment of stem cell niche

**Figure 2:**
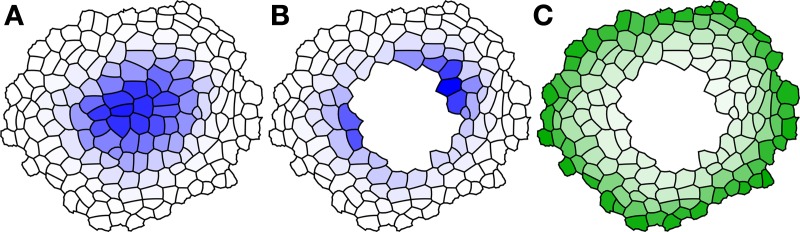
**Results of Wuschel simulation using Cellzilla**. Species concentration is illustrated at the end of the simulation time course. The concentration of Wuschel is shown in blue, and protein Y is shown in green. Darker colors indicate higher concentrations, and white indicates a zero concentration. Steady state concentration of **(A)** Wuschel in wild-type simulation; **(B)** Wuschel in ablated meristem; **(C)** signal protein Y in ablated meristem. Parameters: v = 0.1, T_WY_ = −25, T_WA_ = 0.5, h = 0, k_w_ = 0.1, k_y_ = 0.1, d = 0.5, D_Y_ = 2, D_A_ = 1.5, D_B_ = 15.

In Jönsson et al. (2005a,b) we presented a predictive model of feedback interaction between the Wuschel (WUS) and Clavata3 (CLV) signals in the shoot apical meristem. This simplified model was able both to organize the WUS expression domain and to predict the reorganization due to the removal of the CLV signal from the WUS domain as seen in experiments when cells are ablated. This model uses a reaction-diffusion mechanism to induce WUS; the pattern is induced by a Brusselator. The original model relies on a diffusible parameter *Y* that is produced only in the *L*1 layer of a slice. We present an implementation in which our slice has the L1 layer omitted, and replace this with a boundary condition in which *Y* is held fixed, and allowed to diffuse inward. Assuming the Brusselator is implemented by (30), the Cellzilla network for the WUS activator is given by

(31) $$\begin{array}{l} \{\{\{Y,A\}\mapsto W,GRN[v,\{T_{WY},T_{WA}\},1,h,sigma]\},\\ \;\{W\to \emptyset ,k_{w}\},\{Y\to \emptyset ,k_{y}\},\{A+Y\to Y,d\},\\ \;\{Y\to Y,Diffusion[D_{Y},D_{Y}]\}\} \end{array}$$

where v, T_WY_, T_WA_, h, k_w_, k_y_ and *D*_*y*_ are tunable parameters, and the control word sigma tells Cellerator to replace the usual logistic control function *f*(*x*) = 1/(1 + *e*^−*x*^) with $f(x)=(1+1/\sqrt{1+x^{2}})/2$ (in fact, any monotonic increasing saturating function would work). The results illustrated in Figure 2 show that both the original central maximum (in wild type) and dual, smaller maxima result (in the ablation experiment) as modeled previously. In addition, we show the steady state distribution of the constituent *Y* in Figure 2C, illustrating how it forms a ring in the outer cells abutting L1 and decreasing inward, as desired.

### Growth induced by organizing center

**Figure 3:**
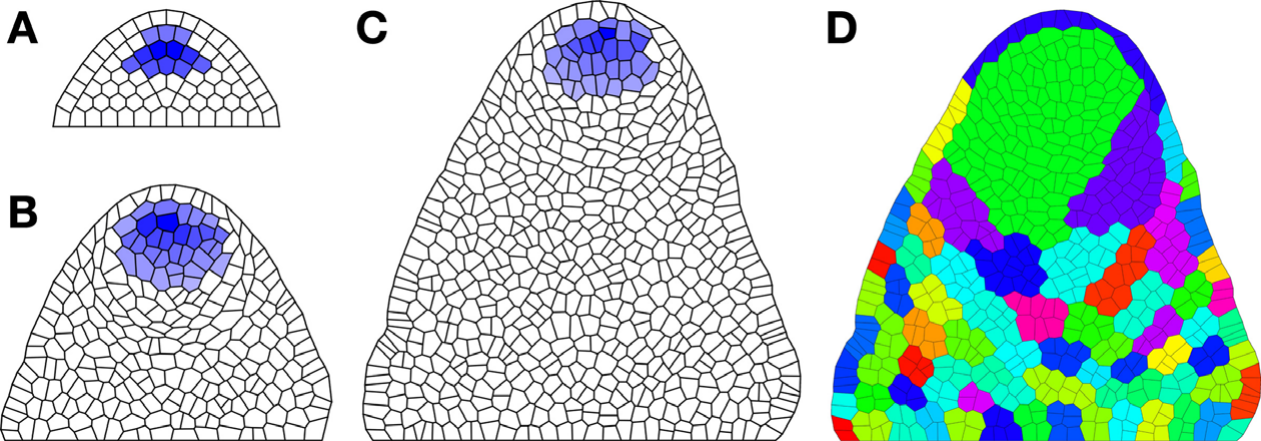
**Maintenance of organizing center during growth simulation. (A)** Initial distribution of *W*; **(B)** Distribution after 250 cell divisions; and **(C)** Distribution after 500 cell divisions. **(D)** Cell lineages; each color shows a different clonal population corresponding to all descendants of a particular cell in **(A)**. Parameters: k_1_ = 2, k_2_ = 0.2, k_3_ = 1, k_4_ = 0.25, k_5_ = 1, k_6_ = 0.05, k_8_ = 1.5, k_9_ = 0.1, D_U_ = 10, D_V_ = 0.5, D_X_ = 0.5, v_X_ = 1, v_W_ = 1, v_V_ = 1, h_X_ = 0, h_W_ = 0, h_V_ = 0, T_WX_ = 4, T_WV_ = 4, T_UW_ = 22.5, T_VW_ = −25, T_WW_ = 27.5, p_0_ = 0.001, p_1_ = 0.004, μ_0_ = 5 × 10^−6^, μ_1_ = .004.

The Brusselator is a useful mathematical/computational artifice for establishing patterns that can be otherwise studied but its biological meaning becomes lost if the variables in the equations do not have biological analogues that are present in the actual tissue. It is more meaningful if the organizing tissue can be established based on specific networks whose constituents have been observed and whose interactions are believed to be present, although this may at times be more computationally intensive. For example, Nikolaev (Nikolaev et al., 2007, 2013) has shown that a combination of reaction-diffusion and feedback in the WUS/CLV network is sufficient to establish a stem cell niche. In a one dimensional dynamic model including cell division, Chickarmane et al. (2012) has shown that negative feedback between WUS and cytokinin synthesis may be sufficient for maintenance of this niche as the tissue grows. Here we present for illustrative purposes of Cellzilla capability a simplified version of a Chickarmane-inspired model, in which two diffusible species are used to establish the pattern: *U* (e.g., that may be part of the cytokinin network), which is produced only in the cells at the tip of the meristem; and a second species *V* that is produced in the L1 layer (e.g., that may produced as part of the CLV network). The species that represents the organizing center is called *W*; *U* and *V* then activate and repress *W*, respectively, while *W* is self-activating, perhaps through an intermediate. Positive feedback of *W* onto *V* is provided by a third diffusible species *X*; and the epidermis is impermeable to *U*, *V* and *W*. Finally, the constitutive degradation of *W* is slightly enhanced in the L2 layer, but occurs everywhere. The network is

(32) $$\begin{array}{l} \{\{\emptyset \to U,k_{1}TIP[t]\},\{U\to \emptyset ,k_{2}\},\{U\to U,Diffusion[D_{U}]\},\\ \{\emptyset \to V,k_{3}L1[t]\},\{V\to \emptyset ,k_{4}\},\{V\to V,Diffusion[D_{V}]\},\\ \{\emptyset ⇄Z,k_{7},k_{8}U[t]\},\{X\mapsto V,GRN[v_{V},T_{WV},1,h_{V}]\},\\ \{\{U,V,W\}\mapsto W,GRN[v_{W},\{T_{UW},T_{VW},T_{WW}\},1,h_{W}]\},\\ \{W\to \emptyset ,k_{6}Z[t]+k_{9}L2[t]\},\\ \{W\mapsto X,GRN[v_{X},T_{WX},1,h_{X}]\},\{X\to \emptyset ,k_{5}\},\\ \{X\to X,Diffusion[D_{X}]\},\\ \{cell\to cell,Grow[GrowthRate[\mu ,f_{\mu }],\\ \text{Pressure[P,f\_P],Spring[k,f\_k]\},}\\ \left\{cell\to cell+cell,Errera[cell,\mu ,\sigma \}\right\} \end{array}$$

where k_1_, k_2_, k_3_, k_4_, k_5_, k_6_, k_7_, k_8_, k_9_, h_V_, h_W_, v_V_, v_W_, T_WX_, T_UW_, T_VW_, T_WW_, T_WV_, D_U_, D_V_, and D_X_ are tunable parameters; L1[t], L2[t], and Tip[t] are built in indicator functions for these cell locations; and the functions f_P_ and f_μ_ describe pressure and growth feedback; e.g., *P*[*i*] = *p*_0_ + *p*_1_*W*[*i*] and μ[*j*] = μ_0_ + μ_1_(*W*[*i*] + *W*[*j*]) (where *p*_0_, *p*_1_, and μ_0_ are tunable control parameters). The user would type these function definitions either in the lines before the model or in line as lambda functions in place of the function references in the model. Simulation results are shown in Figure 3. As seen in Figures 3A–C, the stem cell niche is maintained through at least 500 cell divisions.

## Discussion

We have illustrated that meaningful quantitative results for plant morphodynamics can be obtained using a simple polygonal tissue model coupled with a spring growth equations. In particular, our implementation utilizes and extends an existing arrow-based computational framework that is easy and intuitive to use. This framework is built within a standardized computer algebra system (Mathematica) that is widely available and provides access to a wide selection of analytical tools. In addition we believe that our framework is generalizable and extensible to the wider world or rule-based systems. While more detailed particle-based or molecular dynamics frameworks will certainly produce more accurate frameworks their ultimate extensibility is limited by CPU availability and time constraints. Our implementation provides a useful platform for rapid model development and testing that can easily by transformed to one of the more detailed frameworks, if desired, once suitable results are obtained.

Cellzilla can be used for 2D simulations of plant tissues at the multicellular level. Because models can be rapidly built and tested it allows one to quickly test developmental models both in steady-state and during growth. However, several improvements are planned for future versions. In the current version, only simple transport and diffusion are considered; however, in many cell types, not just plant cells, osmotic and electrical gradients are significant. We plan to implement rules to incorporate both features in future releases. Furthermore, with the addition of osmotic models and pressure gradients we plan to add additional plant-specific growth models (Lockhart, 1965; Ortega, 1985; Geitmann and Ortega, 2009). These can be optionally used in place of the phenomenologically-based spring-based growth model. Additions to the cell division model will be included. For example, the constraints used in the Errera implementation of linear walls and equal areas can be relaxed to quadratic and circular arcs, and the areas can be randomized in a cloud about equality.

A three-dimensional model is also planned, although we expect that this will have significantly greater computational demands. To avoid mathematically unstable solutions in three dimensions the spring model will need to be modified or replaced, most likely with an elastic dynamics model incorporating pressure and stress tensors. An alternative is the use of triangular springs (Delingette, 2008).

### Data sharing

All of the software described here is open source Mathematica (GPL license) and freely downloadable from launchpad at https://launchpad.net/cellerator. The software is fully documented and all examples are available at the project website https://www.cellzilla.info.

### Conflict of interest statement

The authors declare that the research was conducted in the absence of any commercial or financial relationships that could be construed as a potential conflict of interest.

## References

[B1] Andrews S. (2012). Spatial and stochastic cellular modeling with the smoldyn simulator, in Bacterial Molecular Networks: Methods and Protocols: Methods in Molecular Biology, eds van Helden J. Toussaint A. Thieffry D. (New York, NY: Springer), 519–540 10.1007/978-1-61779-361-5_26 22144170

[B2] Besson S. Dumais J. (2011). Universal rule for the symmetric division of plant cells. Proc. Natl. Acad. Sci. U.S.A. 108, 6294–6299 10.1073/pnas.1011866108 21383128 PMC3076879

[B3] Boudin F. Pradal C. Cokelaer T. Prusinkiewicz P. Godin C. (2012). L-Py: an L-system simulation framework for modeling plant architecture development based on a dynamic language. Front. Plant Sci. 3:76 10.3389/fpls.2012.00076 22670147 PMC3362793

[B4] Chickarmane V. S. Gordon V. Tarr S. P. Heisler M. G. Meyerowitz E. M. (2012). Cytokinin signaling as a positional cue for patterning the apicalbasal axis of the growing arabidopsis shoot meristem. Proc. Natl. Acad. Sci. U.S.A. 109, 4002–4007 10.1073/pnas.1200636109 22345559 PMC3309735

[B5] Cickovski T. Aras K. Swat M. Merks R. Glimm T. George H. (2007). From genes to organisms via the cell: a problem-solving environment for multicellular development. Comput. Sci. Eng. 9, 50–60 10.1109/MCSE.2007.74 19526065 PMC2695324

[B6] Delingette H. (2008). Triangular springs for modeling nonlinear membranes. IEEE Trans. Vis. Comput. Graph. 14, 239–241 10.1109/TVCG.2007.70431 18192713

[B7] Errera L. (1888). Ber zellformen und seifenblasen Bot. Centralbl. 34, 395–398 10.1002/cplx.20074

[B8] Faeder J. R. Blinov M. L. Goldstein B. Hlavacek W. S. (2005). Rule-based modeling of biochemical networks. Complexity 10, 22–39 10.1002/cplx.20074

[B9] Faeder J. R. Blinov M. L. Hlavacek W. S. (2009). Rule based modeling of biochemical systems with BioNetGen, in Systems Biology, ed Maly I. V. (New York, NY: Springer), 113–167 10.1007/978-1-59745-525-1_5 19399430

[B10] Geitmann A. Ortega J. K. E. (2009). Mechanics and modeling of plant cell growth. Trends Plant Sci. 14, 1360–1385 10.1016/j.tplants.2009.07.006 19717328

[B11] Gilllespie D. T. (1976). A general method for numerically simulating the stochastic time evlolution of coupled chemical reactions. J. Comput. Phys. 22, 403–434 10.1016/0021-9991(76)90041-3

[B12] Graner F. Glazier J. A. (1992). Simulation of biological cell sorting using a two-dimensional extended potts model. Phys. Rev. Lett. 69, 2013–2016 10.1103/PhysRevLett.69.2013 10046374

[B13] Hucka M. Finney A. Sauro H. M. Bolouri H. Doyle J. C. Kitano H. (2003). The systems biology markup language (SBML): a medium for representation and exchange of biochemical network models. Bioinformatics 19, 513–523 10.1093/bioinformatics/btg015 12611808

[B14] Jönsson H. Heisler M. Reddy G. V. Agrawal V. Gor V. Shapiro B. E. (2005a). Modeling the organization of the wuschel expression domain in the shoot apical meristem. Bioinformatics 21, i232–i240 10.1093/bioinformatics/bti1036 15961462

[B15] Jönsson H. Heisler M. Shapiro B. E. Meyerowitz E. M. Mjolsness E. (2005b). An auxin-drive polarized transport model for phyllotaxis. Proc. Natl. Acad. Sci. U.S.A. 103, 1633–1638 10.1073/pnas.0509839103 16415160 PMC1326488

[B16] Jönsson H. Shapiro B. E. Meyerowitz E. M. Mjolsness E. (2004). Modeling plant development with gene regulation networks including signaling and cell division, in Bioinformatics of Genome Regulation and Structure, eds Hofestaedt R. Kolchanov N. (New York, NY: Kluwer Publications), 311–318

[B17] Karwowski R. Prusinkiewicz P. (2004). The L-System-based plant-modeling environment L-Studio 4.0, in Presented at 4th International Workshop on Functional and Structural Plant Models (Montpelier).

[B18] Koumoutsakos P. Bayati B. Milde F. Tauriello G. (2011). Particle simulations of morphogenesis. Math. Models Methods Appl. Sci. 21(Suppl.), 995–1006 10.1142/S021820251100543X

[B19] Lockhart J. (1965). An analysis of irreversible plant cell elongation. J. Theor. Biol. 8, 264–275 10.1016/0022-5193(65)90077-9 5876240

[B20] Merks R. M. H. Guravage M. Inze D. Beemster G. T. S. (2011). Virtualleaf: an open-source framework for cell-based modeling of plant tissue growth and development. Plant Physiol. 155, 656–666 10.1104/pp.110.167619 21148415 PMC3032457

[B21] Mjolsness E. (2006). The growth and development of some recent plant models: a viewpoint. J. Plant Growth Regul. 25, 270–277 10.1007/s00344-006-0069-7

[B22] Mjolsness E. D. (2013). Time-ordered product expansions for computational stochastic systems biology. Phys. Biol. 10:035009 10.1088/1478-3975/10/3/035009 23735739 PMC3786790

[B23] Mjolsness E. D. Sharp D. H. Reinitz J. (1991). A connectionist model of development. J. Theor. Biol. 152, 429–453 10.1016/S0022-5193(05)80391-1 1758194

[B24] Mjolsness E. D. Yosiphon G. (2006). Stochastic process semantics for dynamical grammars. Ann. Math. Artif. Intell. 47, 329–395 10.1007/s10472-006-9034-121572536

[B25] Nikolaev S. V. Penenko A. V. Lavreha V. V. Mjolsness E. D. Kolchanov N. A. (2007). A model study of the role of proteins CLV1, CLV2, CLV3, and WUS in regulation of the structure of the shoot apical meristem. Russ. J. Dev. Biol. 38, 383–388 10.1134/S1062360407060069 18179025

[B26] Nikolaev S. V. Zubairova U. S. Penenko A. V. Mjolsness E. D. Shapiro B. E. Kolchanov N. A. (2013). Model of structure regulation of stem cell niche in shoot apical meristem of *Arabidopsis thaliana* (in Russian). Doklady Akademii Nauk 453, 336–338 10.1134/S0012496613050104 24150656

[B27] Ortega J. K. E. (1985). Augmented growth equation for cell wall expansion. Plant Physiol. 79, 318–320 10.1104/pp.79.1.318 16664396 PMC1074876

[B28] Plimpton S. J. Slepoy A. (2005). Microbial cell modeling via reacting diffusing particles. J. Phys. 16, 305–309 10.1088/1742-6596/16/1/042

[B29] Pradal C. Dufour-Kowalski S. Boudon F. Fournier C. Godin C. (2008). Openalea: a visual programming and component-based software platform for plant modeling. Funct. Plant Biol. 35, 751–760 10.1071/FP08084 32688829

[B30] Prigogine L. Lefever R. (1968). Symmetry breaking instabilities in dissipative systems. II. J. Chem. Phys. 48, 1695–1700 10.1063/1.1668896

[B31] Prusinkiewicz P. Lindenmayer A. (1990). Algorithmic Beauty of Plants. New York, NY: Springer Verlag

[B32] Prusinkiewicz P. Runions A. (2012). Computational models of plant development and form. New Phytol. 193, 549–569 10.1111/j.1469-8137.2011.04009.x 22235985

[B33] Rudge T. Haseloff J. (2005). A computational model of morphogenesis in plants. Adv. Artif. Life 3630, 78–87

[B34] Sahlin P. Jöhnsson H. (2010). A modeling study of how cell division affects properties of epithelial tissues under isotropic growth. PLoS ONE 5:e11750 10.1371/journal.pone.0011750 20689588 PMC2912771

[B35] Shapiro B. E. Heisler M. Tobin C. Cunha A. Davis A. Mjolsness E. D. (2010). Using geometric markers to predict the cell division plane in meristem cells, in Proceedings of the 6th International Workshop on Functional-Structural Plant Models, eds DeJong T. Da Silva D. (Davis, CA: University of California), 144–146

[B36] Shapiro B. E. Hucka M. Finney A. Doyle J. (2004). MathSBML: a package for manipulating SBML based biological models. Bioinformatics 20, 2829–2831 10.1093/bioinformatics/bth271 15087311 PMC1409765

[B37] Shapiro B. E. Levchenko A. Meyerowitz E. M. Wold B. J. Mjolsness E. D. (2003). Cellerator: extending a computer algebra system to include biochemical arrows for signal transduction simulations. Bioinformatics 19, 677–678 10.1093/bioinformatics/btg042 12651737

[B38] Shapiro B. E. Mjolsness E. D. (2001). Developmental simulations with cellerator, in International Conference on Systems Biology (Pasadena, LA).

[B39] Smith L. G. (2001). Plant Cell Division: Building Walls in the Right Places. Nat. Rev. 2, 33–39 10.1038/35048050 11413463

[B40] Sneddon M. W. Faeder J. R. Emonet T. (2011). Efficient modeling, simulation an dcoarse graining of biological complexity with nfsim. Nat. Methods 8 177–183 10.1038/nmeth.1546 21186362

[B41] Stiles J. R. Bartol T. M. (2001). Monte carlo methods for simulating realistic synaptic microphysiology using MCell, in Computational Neuroscience: Realistic Modeling for experimentalists, ed De Schutter E. (Boca Raton, FL: CRC Press), 87–127

[B42] Yang C.-R. Shapiro B. E. Mjolsness E. D. Hatfield G. W. (2005). An enzyme mechanism language for the mathematical modeling of metabolic pathways. Bioinformatics 21, 774–780 10.1093/bioinformatics/bti068 15509612

[B43] Yosiphon G. (2009). Stochastic Parameterized Grammars: Formalization, Inference, and Modeling Applications. PhD Thesis, UC Irvine.

